# Early treatment with noninvasive positive pressure ventilation prolongs survival in Amyotrophic Lateral Sclerosis patients with nocturnal respiratory insufficiency

**DOI:** 10.1186/1750-1172-4-10

**Published:** 2009-03-10

**Authors:** Pierluigi Carratù, Lucia Spicuzza, Anna Cassano, Mauro Maniscalco, Felice Gadaleta, Donato Lacedonia, Cristina Scoditti, Ester Boniello, Giuseppe Di Maria, Onofrio Resta

**Affiliations:** 1 Institute of Pulmonary Disease, University of Bari, Bari, Italy; 2 Institute of Pulmonary Disease, University of Catania, Catania, Italy; 3 Institute of Pulmonary Disease, University of Naples, Naples, Italy

## Abstract

**Background:**

Amyotrophic lateral sclerosis (ALS) is a neurodegenerative disease, which rapidly leads to chronic respiratory failure requiring mechanical ventilation. Currently, forced vital capacity (FVC) < 50% is considered as physiologic marker for admitting patients to Noninvasive Positive Pressure Ventilation (NPPV) intervention, although it has been recently shown the median survival of patients with baseline FVC < 75% much shorter than median survival of patients with baseline FVC > 75%, independently by any treatment.

**Aim:**

To assess the role of NPPV in improving outcome of ALS, a retrospective analysis was performed to investigate 1 year survival of ALS patients with FVC < 75% and nocturnal respiratory insufficiency, treated with NPPV, compared to a well-matched population of ALS patients, who refused or was intolerant to NPPV.

**Methods:**

We investigated seventy-two consecutive ALS patients who underwent pulmonary function test. Forty-four presented a FVC > 75% and served as control group. Twenty-eight patients presented a FVC < 75% and showed, at polysomnography analysis, nocturnal respiratory insufficiency, requiring NPPV; sixteen were treated with NPPV, while twelve refused or were intolerant.

**Results:**

Increased survival rate at 1 year in patients with FVC < 75% treated with NPPV, as compared to those who refused or could not tolerate NPPV (p = 0.02), was observed. The median rate of decline in FVC% was slower in NPPV patients than in patients who did not use NPPV (95% CI: 0.72 to 1.85; p < 0.0001).

**Conclusion:**

This report demonstrates that early treatment with NPPV prolongs survival and reduces decline of FVC% in ALS.

## Introduction

Amyotrophic lateral sclerosis (ALS) is a neurodegenerative disease characterized by progressive neuromuscular atrophy with early involvement of respiratory system, rapidly leading to pulmonary collapse, which requires mechanical ventilation and represents the major cause of mortality [1]. Over the last decade, noninvasive positive pressure ventilation (NPPV) has been indicated and widely recommended in ALS patients with chronic respiratory failure [2], since not only reduces dyspnoea and improves persistent hypoventilation, [3] but may also extend life of individuals affected by this fatal disease [2,4]. Few patients receive NPPV, although early prediction of respiratory muscle involvement might be useful to plan mechanical ventilation interventions before chronic respiratory failure occurs [5]. Currently, forced vital capacity (FVC) is considered as physiologic marker to admit ALS patients to NPPV treatment [6]. According to previous studies, NPPV should be offered to all subjects with a FVC of less than 50% [7,8]. Recently reports have shown the ultimate role of FVC in ALS patients: a study disclosed that chronic respiratory failure might initiate within 1 year from the first presentation of ALS, in a great proportion of patients, independently of their first respiratory functional status (median FVC% 87) [9], suggesting that the progressive decline of respiratory function might be due to a sudden weakness of respiratory muscles. By the contrast, in a large cohort observation of 1034 patients with ALS, the median survival of patients with baseline FVC < 75% was much shorter than the median survival of patients with baseline FVC > 75% [10], independently by the medical treatment, indicating that a single FVC value is a predictor of survival and disease progression.

Interestingly, neuromuscular deterioration and respiratory decline are also involved in severe sleep-disordered breathing [11] which occurs at an early stage of ALS [12]. Polysomnography analysis reveals impaired nightly sleep hypoventilation, increasing nocturnal oxygen desaturation index, high frequency of apnoea/hypopnea events [13], resulting in nocturnal respiratory insufficiency and decline of cognitive function [14]. Recent studies demonstrated that noninvasive positive pressure ventilation, decreasing sleep-disordered breathing, improves quality of life of patients with ALS [15,16]. These substantiate data address the hypothesis that sleep disturbance together with the impairment of the respiratory functional status are the earliest indication of respiratory insufficiency in ALS patients [17]. In our experience, all patients with FVC < 75% showed nocturnal respiratory disorders.

Aim of the present study was to assess the role of early NPPV treatment in improving outcome of ALS patients. A retrospective analysis was performed to investigate 1 year survival of ALS patients with FVC < 75% and nocturnal respiratory insufficiency, who were treated with NPPV, compared to a well-matched group of ALS patients who did refuse or was intolerant to NPPV.

## Methods

### Study design and population

We investigated seventy-two (43 males) consecutive patients (mean symptom duration at diagnosis was 16.2 ± 5.3 months) who were referred to the pulmonary function and polysomnography laboratories at University of Bari and Catania, from July 2003 to January 2008. Patients were eligible for the analysis if their ages were between 18 and 80 years, had definite or probable diagnosis of ALS. Patients who had other neurological disease, or lung disease unrelated to ALS, were excluded. The protocol was accepted by the local Institutional Review Board. Patients were divided into three groups: forty-four patients (27 males) showed a FVC > 75%, did not undergo polysomnography test, and served as control group (group 1). Twenty-eight patients (16 males) presented a FVC < 75% and nocturnal respiratory insufficiency at polysomnography, requiring NPPV. Sixteen were treated with NPPV (group 2), while twelve refused or were intolerant to NPPV (group 3). Table 1 shows the demographic features and measures of disease severity of the three groups of patients.

**Table 1:** Baseline Characteristics of Patients Entering the Study

	**FVC > 75%**	**FVC < 75%**		***p*-value between groups 2–3**
	Group 1	Group 2 (NPPV)	Group3 (not vent)	
N	44	16	12	
male/female	27/17	9/7	7/5	
Age	51.16 (7.39)	55.93 (5.09)	57.54 (6.54)	ns
BMI	22.72 (3.90)	21.98 (4.48)	22.85 (3.16)	ns
Bulbar onset	13/44	5/16	4/12	ns
Spinal onset	23/44	8/16	6/12	ns
FVC%	83.3 (11.84)	65.13 (13.37)	62.35 (12.75)	ns
FEV1%	88.12 (13.98)	64.53 (14.17)	61.6 (13.8)	ns
PaO2 mmHg	90.35 (9.58)	79.91 (12.48)	77.35 (8.54)	ns
PaCO2 mmHg	36.43 (6.72)	41.83 (9.27)	39.48 (8.92)	ns
ALSFRS-R		28.7 (6.1)	26.7 (7.1)	ns

Data are presented as mean (± SD); ns = not significant

### Amyotrophic Lateral Sclerosis Functional Rating Scale (ALSFRS) and revised ALSFRS (ALSFRS-R)

Disease severity of the patients with FVC < 75% (groups 2 and 3) was measured using the Revised-Amyotrophic Lateral Sclerosis Functional Rating Scale (ALSFRS-R) (Table 1) [18]. ALSFRS is a 10-item functional inventory which was devised for use in therapeutic trials in ALS [19]. Each item is rated on a 0–4 scale, inversely related to disease severity, by the patient and/or caregiver. The ALSFRS assesses patients' levels of self-sufficiency in areas of feeding, grooming, ambulation and communication. The revised ALSFRS-R, ultimately, incorporates three additional assessments including dyspnea, orthopnea, and the need for ventilatory support [18].

### Pulmonary function testing and arterial blood gas analysis

Pulmonary function tests were performed in the pulmonary function laboratory of our Institutes using a spirometer (PK Morgan Ltd; Gillingham, UK). The equipment was calibrated daily using a 3-L syringe and the analysis was performed in accordance to the guidelines of the ATS [20]. The best of three reproducible values was expressed as a percentage of the predicted normal value. To overcome mouth leaks, a full-face mask was adapted for bulbar ALS patients. Patients unable to perform the test were excluded from the study.

Arterial blood for the analysis of gases during room air breathing was drawn with the patient in the supine position, and PaO_2_, PaCO_2 _and pH were measured in a blood gas analyzer (Model 1312; Instrumentation Laboratory; Milan, Italy).

### Sleep study

All subjects of groups 2 and 3 were evaluated in the sleep laboratory of the Institutes of Respiratory Diseases of the Universities of Bari and Catania for one night. They were monitored continuously for 8 hours using a 19-channel polysomnograph (Compumedic; Sydney, Australia). Polysomnography was performed after one night of adaptation in the hospital, according to standard methods[21]. Polysomnography consisted of continuous polygraphic recording from surface leads for electroencephalogram, electro-oculography, electromyography, electrocardiogram, thermistors for nasal and oral airflow, thoracic and abdominal impedance belts for respiratory effort, pulse oximetry for oxyhemoglobin level, and sensor for the position during sleep. Apnoea was defined as complete cessation of airflow lasting ≥ 10 seconds; hypopnea was defined as either a ≥ 50% reduction in airflow for ≥ 10 seconds or a < 50% but discernible reduction in airflow accompanied either by a decrease in oxyhemoglobin saturation of > 4% or an arousal. Severity of nocturnal respiratory insufficiency was assessed by the apnoea-hypopnea index (AHI), mean total sleeping time, and mean arterial oxygen saturation (SpO_2_). Table 2 shows the polysomnographic analysis of the groups 2 and 3.

**Table 2:** Polysomnographic features of the groups 2 (FVC < 75% with NPPV) and 3 (FVC < 75% without NPPV)

**FVC < 75%**	**Group 2 (16) (NPPV)**	**Group 3 (12) (no NPPV)**	***p*-value**
SpO2 per min, (% ± SD)	89 ± 5.2	88 ± 7.4	ns
Total sleep time, min, (mean ± SD)	312 ± 54	306 ± 62	ns
AHI median (range ± SD)	12 ± 7	11 ± 8	ns
AI/h	7 ± 1,38	3.8 ± 6,5	ns
HI/h	17 ± 5,61	19 ± 1.5	ns

Data are presented as mean (± SD); ns = not significant

### Noninvasive ventilation

Patients were prescribed NPPV per standard guidelines, when FVC was less than 75% and nocturnal respiratory symptoms were present. Pressures were routinely begun at 8 cmH_2_O IPAP and 3 cmH_2_O EPAP [22]. The devices used were a volume-controlled ventilator (Life Care Products, Lafayette, Colorado) in assist-control mode or bilevel positive-pressure device (BiPAP, Respironics, Inc., Murrysville, Pennsylvania) in spontaneous-timed mode. Tidal volume (for the volume-controlled ventilator) or pressure (for the bilevel positive-pressure device) were initially adjusted for chest rise, leaks, and patient comfort and were adjusted on subsequent visits to control hypercapnia and dyspnea. Patients were instructed to use noninvasive positive-pressure ventilation nightly as tolerated and as necessary in the daytime, according to previous reports [2,23]. Tolerance was defined as the ability to sleep nightly while receiving noninvasive positive-pressure ventilation for at least 4 consecutive hours.

### Statistical analysis

Data are presented as means ± standard deviation (SD). Unpaired student t-test was used for comparisons between patients treated with NPPV and those who were intolerant or declined NPPV. Survival comparisons were performed by Mantel-Haenszel log-rank test. Pearson correlation coefficients were used to assess the association between the different parameters (Prism vers. 4.0 for Windows). An unpaired *t*-test was used for comparisons between groups for FVC% decline. Significance was established at a p-value < 0.05.

## Results

The mean (SD) age at disease onset in our patient population was 54.9 (6.3) years. As shown in Table 1, there were no statistical significant differences between the groups 2 and 3 of ALS patients regarding sex, age, Body Mass Index (BMI), time from diagnosis, bulbar or spinal onset involvement, FVC%, FEV_1_%, PaO_2_, PaCO_2_, ALSFRS-R questionnaire score and riluzole treatment. No statistical significant differences were observed at polysomnography test in terms of saturation% per minute, total sleep time, and AHI between the group 2 and 3 of patients (Table 2).

**Figure 1:**
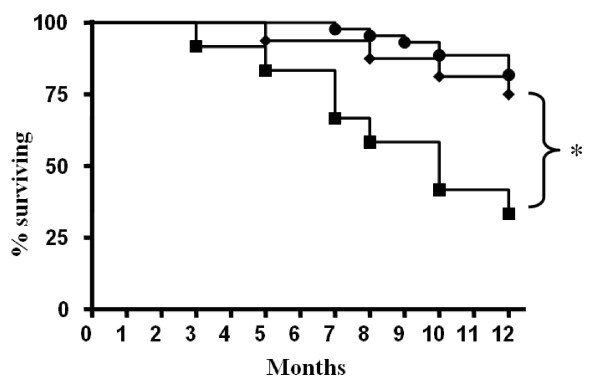
**Kaplan-Meier plots of survival in 72 patients affected by amyotrophic lateral sclerosis (ALS) from the initiation of NPPV**. (Black circle) = 44 patients with ALS with FVC > 75%, (Black triangle) = 16 patients with ALS with FVC < 75% treated with NPPV, (Black square) = 12 patients with ALS with FVC < 75% not treated with NPPV. ALS patients with FVC < 75% treated with NPPV vs. patients with ALS with FVC < 75% intolerant to NPPV: χ^2 ^5.32; *p = 0.02. Patients with ALS with FVC > 75% vs. patients with ALS with FVC < 75% treated with NPPV: χ^2 ^= 0.408 p = 0.5. Patients with ALS with FVC > 75% vs. patients with ALS with FVC < 75% not treated with NPPV: χ^2 ^= 15.4; *p *< 0.0001.

**Figure 2:**
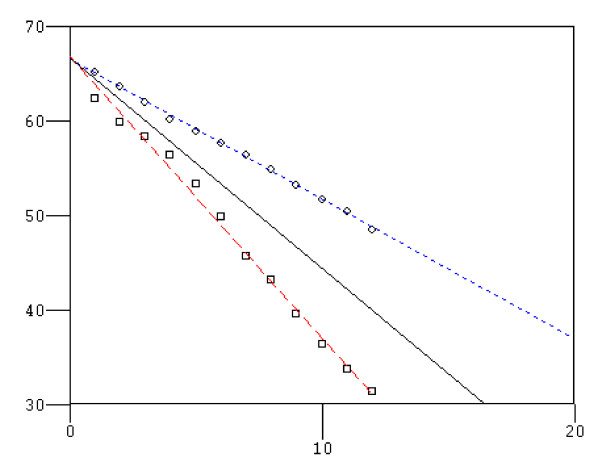
**Slope of FVC% in 1 year between survivors of the groups 2 and 3**. Blue line: Group 2 (12) (NPPV); Red line: Group 3 (4) (no NPPV). X axis: months, Y axis: FVC%. FVC% slope change per month in group 2 (1.52 ± 0.3) and group 3 (2.81 ± 0.8); p < 0.0001.

In Figure 1 is shown the 1 year survival slopes of the groups of ALS patients with FVC > 75%, and of patients with FVC < 75% treated or not with NPPV. Kaplan-Meier 1 year survival rates showed a statistical significant difference between ALS patients with FVC < 75% treated with NPPV and ALS patients with FVC < 75% who refused or were intolerant to NPPV (12/16 vs. 4/12; χ^2 ^= 5.32; p = 0.02), while not statistical significant difference was found between patients with FVC > 75% vs. patients with FVC < 75% treated with NPPV (37/44 vs 12/16; χ^2 ^= 0.408; p = 0.5). Table 3 shows the causes of death of ALS patients with FVC < 75%. Among survivors patients with FVC < 75%, the median rate FVC decline following initiation of NPPV was slower in patients who tolerated NPPV (group 2) than in patients who did not tolerate NPPV (group 3), (Figure 2), FVC% slope change per month was (1.52 ± 0.3) in group 2 and 2.81 ± 0.8 in group 3 (p < 0.0001).

**Table 3:** Causes of death of Patients of groups 2 and 3

	**Causes of death**	
	Group 2 (NPPV) (16)	Group3 (not vent) (12)
Heart failure	1	3
Broncho-Pneumonia and diaphragmatic respiratory insufficiency	2	2
Pulmonary embolism	0	2
Uncertain	1	1

## Discussion

The development of early progressive hypoventilation affects the natural history of ALS, and time for admitting patients to NPPV represents a crucial dilemma for neurologists and pulmonologists. The use of noninvasive positive pressure ventilation has yet been demonstrated to improve survival in ALS patients [2,4,23], although the most useful indicator of chronic respiratory decline and dead risk was previously considered a baseline FVC < 50% [6-8]. Our findings show that ALS patients who receive noninvasive positive pressure ventilation when Forced Vital Capacity at baseline is less than 75% have a significant survival improvement at 1 year, as compared to those, with similar FVC, who refused or can not tolerate NPPV (p = 0.02). In addition, the median rate of FVC decline was slower in survived patients who tolerated NPPV than in patients who were intolerant to NPPV (p < 0.0001). These results are independent by differences in sex, age, BMI, bulbar or spinal onset, pulmonary functions, arterial blood gas analysis, ALSFRS-R questionnaire score, riluzole treatment, and polysomnographic characteristics, including saturation % per minute, total sleep time, and AHI. Recent papers showed contrasting data about disease progression and survival indicators in patients with ALS. While a study established that chronic hypoventilation requiring mechanical ventilation can rapidly occur, in a small number of patients, independently of their initial respiratory function degree (median FVC% 87) [9], a recent paper revealed the FVC value > 75%, as an early positive predictor of survival in a large number (1034) of ALS patients[10]. Very lately, in a randomised controlled trial, Bourke and co-workers showed that application of noninvasive ventilation when orthopnea occurred, associated to reduction of maximal inspiratory pressure < 60% of the predicted, improved survival, as compared to standard care, in ALS patients [24]. Our data, already published as preliminary results [25], are similar to the findings of Lechtzin and colleagues, who admitted ALS patients with FVC < 65% to mechanical ventilation and observed a significant prolongation of survival [26].

Our study provides additional data about the role of sleep disorders, which actively participate to respiratory failure in ALS. Indeed, the respiratory failure may be present in the absence of breathlessness at rest, or orthopnoea [27], while, it has been observed that sleep disturbance appears at an early stage of disease [12], when respiratory muscle weakness is not sufficient to cause daytime orthopnea. Sleep-disordered breathing might be likely the earliest indication of respiratory insufficiency [17]. In addition, in ALS patients with nocturnal insufficiency, NPPV has been demonstrated to correct sleep-disordered breathing, enhancing quality of life[16].

This study supports the hypothesis that NPPV should be immediately prescribed to ALS patients with mild respiratory dysfunction (FVC < 75%) and polysomnographic signs of nocturnal hypoventilation, for at least 4 hours per day, in order to delay the rapid progression toward chronic respiratory failure. In particular, NPPV treatment significantly decreased the mortality rate of ALS patients with FVC < 75%, as well as the median FVC% rate decline, resulting much slower when compared to the slope of vital capacity of ALS patients with FVC < 50%, treated with 4 hours per day with bi-level intermittent positive pressure, as earlier reported [23].

Although in the present study, the small number of patients treated with noninvasive mechanical ventilation can not give significance for a definitive conclusion, our findings encourage the early use of NPPV, in order to extend survival and to reduce the decline of lung volumes and compliance, thus ameliorating the respiratory function and quality of life of these patients.

In conclusion, this preliminary report demonstrates that early treatment with NPPV prolongs survival in ALS patients, indicating for the first time that NPPV should be introduced when FVC drops below 75% and not 50%, as considered standard care for these patients previously, although further multicentric studies must be conducted to well establish it.

## Abbreviations

ALS: Amyotrophic lateral sclerosis; ALSFRS-R: Amyotrophic lateral sclerosis functional rating scale – revised; AHI: apnea-hypopnea index; Bi-level: bilevel positive airway pressure; BMI: Body Mass Index; FEV_1_: Forced Expiratory Volume in the first second; FVC: Forced Vital Capacity; NPPV: Noninvasive Positive Pressure Ventilation; SpO_2_: Mean arterial oxygen saturation.

## Competing interests

The authors declare that no significant conflicts of interest exist with any companies/organization whose products or services may be discussed in this article.

## Authors' contributions

PC designed the study, carried out the laboratory research and the patients' characterisation for the classification of the different patient groups, and wrote the manuscript. LS designed the study, recruited the patients, coordinated the study, and assisted in performing the statistical analysis. AC designed the study, recruited the patients, and coordinated the study. MM performed statistical analysis and interpretation of results. FG recruited the patients and helped with study design. DL performed the statistical analysis and participated in reviewing the manuscript. CS assisted in the patients' characterisation and in statistical analysis. EB recruited the patients and helped with study design. GDM participated in interpretation of results and critically reviewed the manuscript. OR conceived and supervised the study as head of the lung research group, participated in its design and coordination and revised the manuscript. All authors read and approved the final manuscript.
